# Nano ist groß! Fakten und Mythen über nanopartikuläre UV-Filter

**DOI:** 10.1007/s00105-022-04954-1

**Published:** 2022-02-21

**Authors:** Christian Surber, James Plautz, Uli Osterwalder

**Affiliations:** 1 Dermatologische Klinik, UniversitätsSpital Zürich, Gloriastr. 31, 8091 Zürich, Schweiz; 2 Dermatologische Klinik, Universitätsspital Basel, Petersgraben 4, 4031 Basel, Schweiz; 3 CHRYSALIS Services AG, Bäumleingasse 10, 4051 Basel, Schweiz; 4 Sun Protection Facilitator GmbH, Pfeffingerstr. 82, 4053 Basel, Schweiz

**Keywords:** Nanopartikel, Sonnenschutzmittel, UV-Filter-Sicherheit, EU-Verordnungen, Sonnenschutzmittelformulierung, Nanoparticles, Sunscreen products, UV filter safety, EU regulations, Sunscreen formulations

## Abstract

Seit den 1950er-Jahren sind anorganische Sonnenschutzmittel mit mikronisiertem Titandioxid (TiO_2_) und Zinkoxid (ZnO) erhältlich. Deren kosmetische Akzeptanz blieb beschränkt, da sie als weiße Paste auf der Haut zurückbleiben. Durch Verkleinerung der Partikel in den Nanobereich unter 100 nm wurde deren optische Eigenschaft, sichtbares Licht zu reflektieren, verringert. Nach 2000 wurden auch organische Filter in dieser Größenordnung entwickelt. Die damals herrschende Begeisterung für Nanotechnologie übertrug sich nicht auf Sonnenschutzmittel mit nanopartikulären Filtern. Verbraucher vermuten, dass die Partikel durch die Haut dringen, vom Blut aufgenommen werden, sich im Körper verteilen und Krankheiten verursachen. Nicht zuletzt aufgrund von Druck der Öffentlichkeit wurden Kosmetika – zu denen auch Sonnenschutzmittel gehören – das erste Produktsegment, in dem nanopartikuläre Stoffe strengen Regeln unterworfen wurden. Trotz fortschrittlicher Regulierung und strenger Zulassungsverfahren für nanopartikuläre Filter blieben Vorbehalte bestehen. Mögliche Gründe dafür sind mangelnde Kenntnisse über die geltenden Rechtsvorschriften oder Misstrauen gegenüber diesen, unklare Vorstellungen über das Verhalten von Nanopartikeln in Sonnenschutzmitteln und infolgedessen eine unklare Wahrnehmung von Gefahr, Risiko und Exposition. Vor diesem Hintergrund werden das Wesen und Verhalten von nanopartikulären Filtern in Sonnenschutzmitteln auf der Haut und potenziell in der Haut sowie der Regulierungsrahmen, der sicherstellt, dass nanopartikuläre Filter und die sie enthaltenden Sonnenschutzmittel sicher in der Anwendung sind, diskutiert.

## Abstract (en)

Sunscreen products containing inorganic micronized titanium dioxide (TiO_2_) and zinc oxide (ZnO) have been available since the 1950s. Their cosmetic acceptance remained limited as they persist as a white paste on the skin. By reducing the size of the particles into the nano-range below 100 nm, their optical property of reflecting visible light is reduced. After the year 2000, organic filters of this size range were developed. The enthusiasm for nanotechnology that prevailed at the time did not transfer to sunscreen products with nanoparticulate filters. Consumers suspect that the particles permeate the skin, are absorbed by the blood, and spread throughout the body causing illness. Not least due to public pressure, cosmetics—which include sunscreen products—became the first product segment in which accordingly manufactured substances were subjected to strict regulations. Despite advanced regulation and strict approval procedures for nanoparticulate filters, public reservations remained. Possible reasons for this are lack of knowledge or mistrust of the applicable legislation, unclear perception of the behavior of nanoparticles in sunscreen products and as a result unclear perceptions of hazard, risk, and exposure. Against this background, the nature and behavior of nanoparticulate filters in sunscreens on the skin and potentially in the skin, as well as the regulatory framework that ensure that nanoparticulate filters and the products containing them are safe to use are discussed.

Nano ist eine Metapher für etwas besonders Kleines. Bei Sonnenschutzmitteln, die nanopartikuläre UV-Filter enthalten, hat diese Vorstellung die Befürchtungen hervorgerufen, dass solche Filter besonders leicht von der Haut aufgenommen werden und unerwünschte Wirkungen hervorrufen. Bei genauer Betrachtung aller zugelassenen Filter wird jedoch ersichtlich, dass nanopartikuläre Filter um ein Vielfaches größer sind als alle nichtpartikulären (löslichen) Filter. Interzellulärlipide und Korneodesmosomen des Stratum corneum verhindern die Diffusion nanopartikulärer Filter, während sie lösliche Filter wenig behindern. Nanopartikuläre Filter sind eine Alternative zu den viel kleineren löslichen Filtern.

Pigmenthaltige Präparate werden seit dem Altertum zum Schutz vor den negativen Auswirkungen der Sonnenstrahlung verwendet. Um 1950 erwähnte die US Food and Drug Adminstration (FDA) erstmals Titandioxid (TiO_2_) im Zusammenhang mit einem Sonnenschutzmittel – später auch Zinkoxid (ZnO). Die mittlere Partikelgrößenverteilung von TiO_2_ und ZnO in diesen Produkten war dergestalt, dass das Licht hauptsächlich gestreut oder reflektiert wurde, wodurch die Haut physikalisch vor UV-Strahlung geschützt wurde. Diese Art des Schutzes führte dazu, dass sie als physikalische Filter (positive Konnotation) bezeichnet wurden. Während deren anorganische Eigenschaften sie von allen anderen Filtern unterschied, die aus organischen Ausgangsmaterialien synthetisiert wurden, bezeichnete man sie fälschlicherweise als chemische Filter (negative Konnotation). Diese Unterscheidung ist irreführend. Alle Filter sind chemische Substanzen, und die Wirkweisen – Absorption, Reflexion und Streuung – stellen physikalische Phänomene dar. Obwohl korrekt, ist die Unterscheidung zwischen anorganischen und organischen Filtern unpraktisch, da sie für die Beurteilung kaum relevant ist und die Tatsache nicht berücksichtigt, dass sowohl bei anorganischen wie auch bei organischen Filtern partikuläre Formen existieren. Ein für die Entwicklung von Sonnenschutzformulierungen und die toxikologische Beurteilung von Filtern relevantes Kriterium ist die Unterscheidung zwischen löslichen und unlöslichen Filtern. Löslich heißt in diesem Kontext, dass die Filter in den Ingredienzien der Sonnenschutzformulierung molekulardispers gelöst und verteilt sind, während unlöslich bedeutet, dass die Filter in der Sonnenschutzformulierung als multimolekulare Agglomerate bzw. Partikel dispergiert sind [5, 31].

Für die toxikologische Beurteilung ist die Unterscheidung zwischen löslichen und unlöslichen Filtern wichtig

Die ersten kommerziell erhältlichen Sonnenschutzmittel, die TiO_2_ oder ZnO enthielten, wurden bald als kosmetisch unattraktiv angesehen, da sie aufgrund der Partikelgröße der Filter als weiße Paste auf der Haut sichtbar blieben. Um diese ungünstige Eigenschaft zu beseitigen, wurde die Partikelgrößenverteilung in einen Bereich unter 100 nm gesenkt, ein Schwellenwert, der die optische Eigenschaft der Partikel, sichtbares Licht zu reflektieren (380–780 nm), verringert. Vorausgesetzt, dass die kleineren Partikel gut dispergiert sind und nicht agglomerieren, werden die Sonnenschutzmittel weitgehend transparent. Darüber hinaus wird ihre Fähigkeit, vor UV-B- und UV-A-Strahlung (280–400 nm) zu schützen, noch verstärkt.

Mitte der 1990er-Jahre gab es die ersten Sonnenschutzmittel mit nanopartikulärem TiO_2_ und ZnO. Mit der rasanten Entwicklung der Nanotechnologie nach der Jahrtausendwende wurden auch neue nanopartikuläre organische Filter entwickelt. Damals nährte die Nanotechnologie große Hoffnungen in den Fortschritt von Wissenschaft und Technik, auch im Gesundheitssektor und in der Kosmetikindustrie. Allerdings bald, beeinflusst durch Bilder aus der Science-Fiction-Literatur von sich selbst replizierenden Nanorobotern, die alles Lebendige zerstören, oder von Gesundheits- und Umweltkatastrophen, die durch Asbest verursacht wurden, wurde die Akzeptanz für Produkte mit Nanomaterialien in breiten Bevölkerungskreisen beeinträchtigt. Eindrucksvolle Hinweise dafür sind Schlagzeilen in der Presse, die die Begriffe wie „Nanoangst“ oder „Nano – das neue Asbest“ hervorbrachten. Die Begriffe sind zu starken Frames geworden, die die Bedenken in der Öffentlichkeit fast automatisch verstärken. Der Begriff „Nanoangst“ scheint sich im Bewusstsein der Bevölkerung festgesetzt zu haben, hervorgerufen, verstärkt und aufrechterhalten durch uninformierte Meinungen und sensationsheischende Schlagzeilen, die die kleinen Partikel als eine inhärente Gefahr darstellen. Verbraucher vermuten, dass die Nanopartikel durch die Haut dringen, vom Blut aufgenommen werden und sich im ganzen Körper verteilen und Krankheiten verursachen [8, 33]. Das jüngste Beispiel, das sich dieser Befürchtung bedient, ist der letzte Bond-Film („Keine Zeit zu sterben“, 2021)! Bond opfert sich, um seine Freundin und ihre Tochter vor dem Nanobot-Virus zu schützen, mit dem er infiziert wurde.

Leider wird in der wissenschaftlichen Literatur nicht angemessen auf diese Befürchtungen eingegangen, da sie Unsicherheiten oft stilisiert ausdrücken, die zwar wissenschaftlich vertretbar sind, wenn sie sich an ein Fachpublikum richten, den Laien oder Verbraucher aber verunsichern. Beispiele aus Schlussfolgerungen illustrieren dies: Nanopartikel in Sonnenschutzmitteln „… können eine *Gefahr* darstellen (Potenzial Schäden zu verursachen), und weitere Studien sind erforderlich …“; oder umgekehrt „… stellen kein oder ein vernachlässigbares *Risiko* (Wahrscheinlichkeit eines Schadens bei Exposition) für die menschliche Gesundheit dar, bieten aber große gesundheitliche Vorteile wie …“ usw. Der Unterschied zwischen Gefahr, Risiko und Exposition wird nicht immer klar kommuniziert oder richtig verstanden.

Risiko ist die Wahrscheinlichkeit eines Schadens aufgrund einer Exposition

In der Europäischen Union (EU) sind Sonnenschutzmittel als Kosmetika reguliert [34] Nicht zuletzt aufgrund des öffentlichen Drucks wurden Kosmetika das erste Produktsegment weltweit, in dem kosmetische Fertigprodukte mit Nanomaterialien strengeren Regeln unterworfen wurden. Trotz fortschrittlicher Regulierung und strenger Zulassungsverfahren bestehen nach wie vor weitverbreitete Vorbehalte gegenüber Sonnenschutzmitteln, die Nanopartikel enthalten. Mögliche Gründe dafür sind mangelnde Kenntnisse über die geltenden Rechtsvorschriften oder Misstrauen gegenüber diesen, unklare Vorstellungen über das Verhalten von Nanopartikeln in Sonnenschutzmitteln und infolgedessen eine unklare Wahrnehmung von Gefahr, Risiko und Exposition. Leider sind viele Angehörige der Gesundheitsberufe heute nicht in der Lage, Patienten und Verbrauchern diese Fakten zu erklären.

Vor diesem Hintergrund wird in diesem Beitrag Folgendes erläutert:

- die Beschaffenheit und das Verhalten von nanopartikulären Filtern in Sonnenschutzformulierungen, auf der Haut und potenziell in der Haut,
- der Rechtsrahmen, der sicherstellt, dass nanopartikuläre Filter und die sie enthaltenden Sonnenschutzmittel in der Anwendung sicher sind,
- die Begrifflichkeit „Gefahr, Risiko und Exposition“ im Kontext von Sonnenschutzmittel sowie
- der Einfluss von wissenschaftlichen Veröffentlichungen, Science-Fiction-Geschichten und Medien auf die Wahrnehmung.

## Das Wesen partikulärer UV-Filter

Die Tab. 1 stellt alle partikulären (unlöslichen) Filter mit EU-Zulassungen bzw. Scientific Committee on Consumer Safety(SCCS)-Stellungnahmen vor.

**Table 1:** 

*Anorganische UV-Filter,* International Nomenclature of Cosmetic Ingredients (INCI)
– Titanium Dioxide (nano), maximale Einsatzkonzentration (mEk) 25 % [24]
– Zinc Oxide (nano) mEk 25 % [23]
*Organische UV-Filter,* INCI
– Methylene Bis-benzotriazolyl Tetramethylbutylphenole (nano) (MBBT) mEk 10 % [25]
– Tris-biphenyl Triazine (nano) (TBPT) mEk 10 % [22]
– Phenylene Bis-diphenyl Triazine (PBDT) mEk 5 % [19]
– Bis-(Diethylaminohydroxybenzoyl Benzoyl) Piperazine (nano) (HAA299) mEk 10 % [20]

### Quelle und Produktion

Die anorganischen Filter TiO_2_ und ZnO werden aus den metallhaltigen Erzen Ilmenit (Manakanit) und Zinkblende (Sphalerit) unter Verwendung konzentrierter Schwefelsäure bzw. Chlorgas gewonnen. Vor diesem Hintergrund ist das für diese Filter oft verwendete Prädikat natürliche Filter irreführend. Die Filter werden in partikulärer und nanopartikulärer Form eingesetzt. Nanopartikel sind oft beschichtet, um einerseits deren Einarbeitung und Verteilbarkeit in Formulierungen zu erleichtern und andererseits die oxidationskatalytischen Eigenschaften insbesondere von TiO_2_ unter UV-Bestrahlung zu verhindern. Die Beschichtung erfolgt mit anorganischen (Aluminium‑, Siliziumoxid) oder organischen Verbindungen (Fettsäuren, Silikonen). Die organischen Filter sind Produkte mehrstufiger Syntheseprozesse. Sie werden in Nassmahlverfahren auf bestimmte Partikelgrößen gemahlen und mit einer Partikelgrößenverteilung von 40–120 nm oder größer in Aufschlämmungen eingesetzt [5, 31].

### Abmessungen und physikalische Integrität

Sowohl in der Wissenschaft als auch im Alltag werden die Begriffe „Nano“ und „Mikro“ häufig verwendet, ohne die Dimensionen näher zu definieren. Die Grenze zwischen dem Nano- und Mikrobereich ist oft willkürlich und wird je nach Anwendungsumfeld unterschiedlich interpretiert. In der Verordnung (EG) Nr. 1223/2009 über kosmetische Mittel, Artikel 2 Absatz 1 Buchstabe k bezeichnet der Begriff „Nanomaterial“ ein unlösliches oder biobeständiges und absichtlich hergestelltes Material mit einer oder mehreren äußeren Abmessungen oder einer inneren Struktur in der Größenordnung von 1–100 nm [34]. Für Nanomaterialen, die ihre Nanostruktur verlieren – z. B. in einer Formulierung oder an einer biologischen Umgebung, aufgrund von Solubilisierung oder Abbau –, gilt die genannte Definition nicht (z. B. Liposomen, Nanoemulsionen).

Nanopartikel im Sinne der Regulierung sind unlöslich oder biobeständig und 1–100 nm groß

Verschiedene Regulierungsbehörden, Industrie und Normungsorganisationen haben die Definition erweitert und präzisiert [27]. Die unterschiedliche Ausrichtung der Definitionen kann gelegentlich zu inkonsistenter Identifizierung und Bewertung von Nanomaterialien führen und sich nachteilig auf die wissenschaftliche Bewertung und die öffentliche Wahrnehmung der Nanotechnologie auswirken. Ein aktuelles Beispiel ist die Löslichkeit von ZnO-Nanopartikeln im leicht sauren pH-Bereich der Haut. Nach den Definitionen des SCCS bzw. des Europäischen Arzneibuchs ist ZnO unlöslich bzw. praktisch unlöslich (≤0,1 g/l) (Tab. 2 in [21], S. 16). Mit geeigneter Analytik können allerdings gelöste Zn-Ionen in Mengen nachgewiesen werden, die unterhalb der oben genannten Definition von Löslichkeit liegen. Darüber hinaus sind gelöste Zn-Ionen bereits im menschlichen Organismus vorhanden. Deren Konzentration wird im Körper ständig ausgeglichen (Zn-Homöostase) und stellt deshalb kein Problem dar. Nanopartikel weisen einige physikochemische Besonderheiten auf. Mit abnehmender Partikelgröße nimmt das Verhältnis von Oberfläche zu Volumen geometrisch zu. Wenn die Partikeloberflächen biologisch reaktiv sind, steigt die kollektive Reaktivität des Nanomaterials pro Masseneinheit ebenfalls geometrisch an (z. B. Bildung reaktiver Sauerstoffradikale). Diese Phänomene werden durch die genannte Oberflächenbeschichtung und den Zusatz von Antioxidanzien zur Sonnenschutzformulierung verhindert. Anforderungen an Beschichtungseigenschaften von TiO_2_ und ZnO sind ebenfalls reguliert [26].

### Schutzbereich und Wirkungsweise

**Figure 1:**
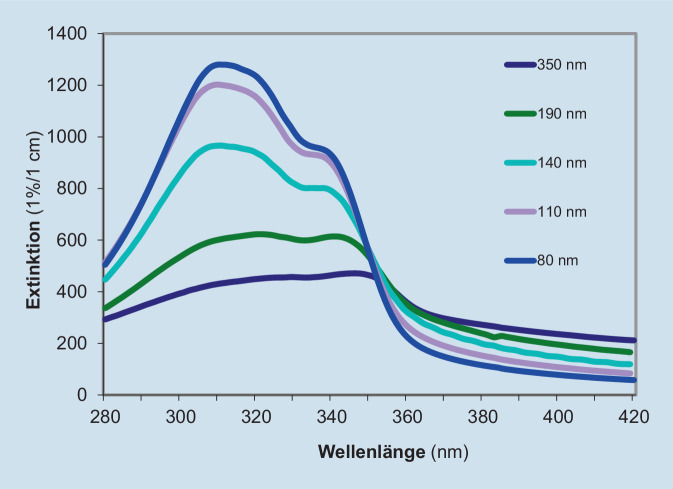

Nanopartikuläre Filter bieten Schutz in einem breiten Bereich des UV-Spektrums. Wirkungsweise und Ausmaß des Schutzes – abhängig von der Wellenlänge und Partikelgröße – sind Absorption, Streuung und Reflexion (Abb. 1; [11]). Derzeit werden nanopartikuläre Filter mit löslichen Filtern kombiniert verwendet. Dies hat den Vorteil, dass die Fähigkeit der Partikel, Strahlung zu streuen, den Wirkungsgrad gelöster Filter erhöht (Erhöhung der Anzahl Kontakte mit dem Photon) [12].

## Dermale Exposition

Die Haut verhindert das Eindringen von Xenobiotika und Fremdkörpern wie Viren und Bakterien. Dennoch ist sie nicht völlig undurchlässig. Die perkutane Absorption einer Substanz ist abhängig von den physikochemischen Eigenschaften der Substanz (Filter) selbst, seines Vehikels (Sonnschutzmittelformulierung) und dem Hautzustand.

### Substanz

**Figure 2:**
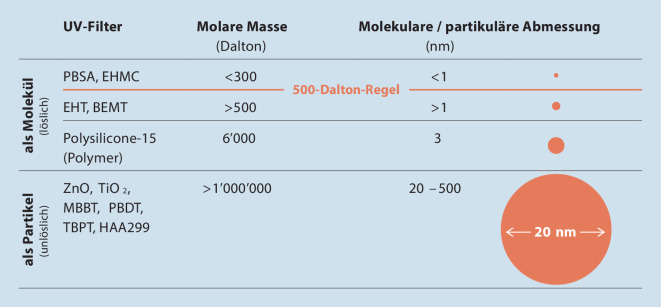

**Figure 3:**
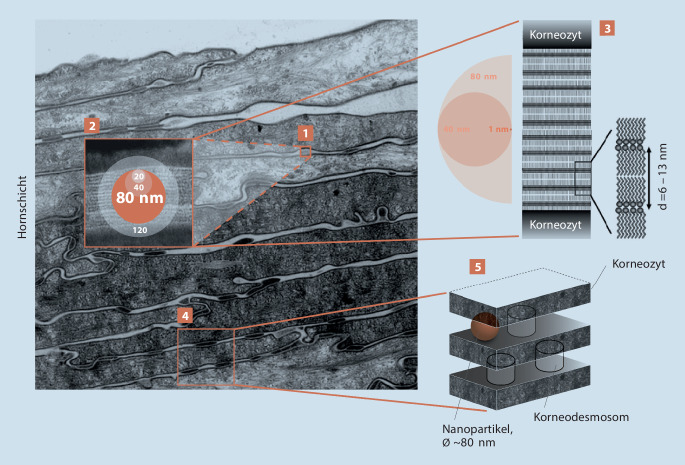

Zu den wichtigsten Substanzeigenschaften gehören Molekulargewicht und Polarität. Die Abb. 2 zeigt Molekulargewichte bzw. Partikeldurchmesser von Filtern. Die Polarität ist ebenfalls wichtig für die Abschätzung einer möglichen perkutanen Absorption. Für Substanzen mit einem logP_Octano/Wasser_-Verteilungskoeffizient von ≤-1 (hydrophil) bzw. ≥4 (lipophil) ist eine perkutane Absorption gering bis unwahrscheinlich [21]. Die histologische Situation in der Haut muss ebenfalls berücksichtigt werden (Abb. 3).

### Vehikel

**Figure 4:**
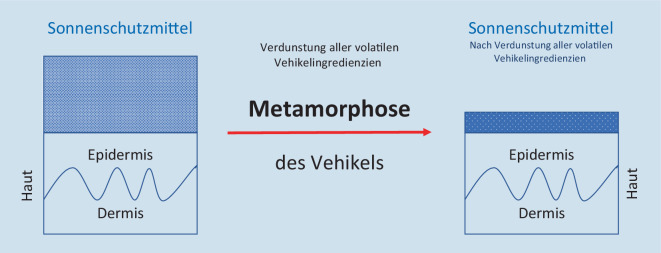

Das Vehikel kann zum einen als Format (Creme, Gel) und zum anderen als Summe seiner Vehikelingredienzien definiert werden. Nach dem Auftragen verändert sich das Vehikel des Sonnenschutzmittels (Abb. 4; [30]).

### Hautzustand

Von besonderem Interesse ist die Frage nach der Anwendung von Sonnenschutzmittel auf geschädigter oder kranker Haut. Dies gilt für enthaarte, rasierte, gereizte oder sonnenverbrannte Haut oder für Haut, die zu Atopie oder Akne neigt. Oft geht man davon aus, dass kranke Haut viel durchlässiger für topisch aufgetragene Substanzen ist. Während dies für einige wenige Substanzen wie Wasser, 5‑Fluoruracil oder Triamcinolonacetonid bestätigt wurde, ist die Absorption der meisten untersuchten Substanzen durch klinisch kranke Haut im Vergleich zu intakter Haut nur in geringem Maße erhöht. Aufgrund der begrenzten Datenlage bleiben Antworten zu dieser Frage weiterhin vage [4, 17].

### Studien zur Absorption von Nanomaterialien

Im Mittelpunkt des Interesses steht die Frage, ob Nanopartikel die Haut durchdringen können. Es gibt Berichte, die eine Aufnahme von Nanopartikeln durch die Haut für unwahrscheinlich halten, und andere, die eine Aufnahme vermuten und ebenfalls mit Experimenten zu belegen versuchen [13, 16]. Die Auswertung solcher Studien ist eine Herausforderung, da die Versuchsbedingungen oft unterschiedlich sind. Die dänische Umweltschutzbehörde und die Europäische Chemikalienagentur (ECHA) stellen in kritischen Übersichten fest, dass der größte Teil der veröffentlichten Arbeiten zur Absorption von Nanomaterialien nicht den heutigen Qualitätsanforderungen genügen [7, 9].

Der Mangel an Studien mit validierter und standardisierter Methodik zur Messung der Absorption von Nanomaterialien schränkt die Schlussfolgerungen eines großen Teils der Untersuchungen ein. Die SCCS hat deshalb Dokumente erarbeitet, die die Charakterisierung der Nanopartikel spezifiziert und den Rahmen beschreibt, in denen die Experimente durchgeführt werden sollten [27]. Zurzeit liegen keine Studien vor, die den Nachweis erbringen, dass nanopartikuläre Filter die menschliche Hautbarriere durchdringen und als Partikel systemisch verfügbar wurden. Die Abb. 2 und 3 zeigen deutlich, dass unlösliche Filter – aufgrund ihrer Größe – kaum ins Stratum corneum eindringen können.

## Rechtlicher Rahmen

### Regulierung von Sonnenschutzmitteln

In der EU sind Sonnenschutzmittel als „Kosmetika“ im Rahmen der Verordnung (EG) Nr. 1223/2009 über kosmetische Mittel reguliert (Art. 2 Abs. 1 a) [34]. In den USA oder Australien sind sie als Arzneimittel reguliert. Kosmetische Fertigerzeugnisse enthalten unzählige Substanzen. Da diese auf biologische Prozesse des Körpers negative Wirkungen haben können, ist eine begrenzte Zahl solcher Substanzen auf der Grundlage ihres Sicherheitsprofils in Anhängen der Verordnung reguliert. Zu diesen Substanzen gehören neben Konservierungsmitteln und anderen Substanzen auch Sonnenschutzfilter. Letztere sind im Anhang VI gelistet. Hier sind u. a. maximale Einsatzkonzentrationen oder nichtzulässige Anwendungsarten festgelegt (sprühbare Formen).

Die Sicherheit kosmetischer Fertigerzeugnisse wird auf 2 Ebenen adressiert:

- Die Sicherheit der UV-Filter muss durch deren Hersteller garantiert werden, und der Filter muss durch die Behörden zugelassen sein.
- Die Sicherheit aller anderen Substanzen, die im kosmetischen Fertigerzeugnis enthalten sind, muss durch das Unternehmen, das das Fertigerzeugnis vertreibt, garantiert werden.

### Zulassung von UV-Filtern

Ähnlich wie bei der Arzneimittelzulassung werden auch UV-Filter nach behördlicher Prüfung auf Sicherheit und Eignung für deren Verwendung zugelassen. Filterhersteller müssen die Ergebnisse spezifischer Sicherheitsprüfungen (Gefährdungsbeurteilung) und Sicherheitsbewertungen (Risikobewertung) für jeden Filter den EU-Behörden zur Prüfung und Bewertung durch den „Wissenschaftlichen Ausschuss für Verbrauchersicherheit“ (Scientific Committee on Consumer Safety [SCCS]) vorlegen [28]. Es handelt sich um einen Ausschuss unabhängiger wissenschaftlicher Sachverständiger in den Bereichen Toxikologie, Chemie usw., die die EU-Kommission in Form von Stellungnahmen (Opinions) zu spezifischen Fragen aller Arten von Gesundheits- und Sicherheitsrisiken bei allen Non-Food-Konsumgütern einschließlich Kosmetika berät. Das formale Verfahren für die Aufnahme eines Filters in Anhang VI umfasst eine erste Einreichung des Dossiers bei der Generaldirektion (GD) Binnenmarkt, Industrie, Unternehmertum und KMU (GD GROW) und der GD Gesundheit und Lebensmittelsicherheit (GD SANTE) der EU-Kommission. Diese geben dem SCCS ein Mandat zur Erstellung einer wissenschaftlichen Stellungnahme über die Sicherheit des Filters. Eine Arbeitsgruppe innerhalb des SCCS prüft die Daten, erstellt den Entwurf einer Stellungnahme zur Prüfung durch den gesamten SCCS und sendet ihn an den Hersteller, die Vertreter der GD sowie an die SCCS-Webseiten zur öffentlichen Prüfung. Nach einer öffentlichen Überprüfung werden alle öffentlichen Stellungnahmen und die Daten vom SCCS erneut geprüft und in einer endgültigen Stellungnahme zusammengefasst. Stuft die SCCS den Filter als sicher ein, wird er in Anhang VI aufgenommen, wenn die EU-Kommission und die Mitgliedstaaten die Stellungnahme des SCCS akzeptiert haben. Daraufhin wird die Kosmetikverordnung durch die Veröffentlichung des überarbeiteten Anhangs VI im EU-Amtsblatt geändert [1]. Erst dann darf der UV-Filter den kosmetischen Fertigprodukten zugefügt werden.

Europa verfügt über eine moderne Regulierung, die die Sicherheit von UV-Filtern garantiert

Da viele der Filter in Mengen von über 1000 kg pro Jahr hergestellt werden, sind vom Hersteller auch Auflagen der Europäischen Chemikalienagentur (European Chemical Agency [ECHA]) zu erfüllen [10]. Die Hersteller müssen nachweisen, wie die Substanz, ggf. deren Nanoform, sicher verwendet werden kann.

### Sicherheitsanforderungen an UV-Filter

Informationen zu und Anforderungen an die Sicherheitsprüfung für UV-Filter sind in den „SCCS Notes of Guidance for the Testing of Cosmetic Ingredients and their Safety Evaluation“ sowie in den „SCCS Guidance on the Safety Assessment of Nanomaterials in Cosmetics“ aufgeführt [21]. Folgende Informationen werden benötigt: physikalische und chemische Charakterisierung einschließlich Reinheitsdaten; akute, subchronische, Photo- und Photogenotoxizität, dermale Absorption, Hautreizung, Schleimhautreizung sowie Hautsensibilisierung. Unter Umständen können zusätzliche Tests zur Bewertung der Toxikokinetik, der Entwicklungs- und Reproduktionstoxizität, der Karzinogenität oder der Genotoxizität erforderlich sein. Bei Nanomaterialien sind zusätzlich Informationen wie Partikelgröße, -größenverteilung, -form -oberfläche, -oberflächenbehandlung sowie Löslichkeit, Agglomerations- und Aggregationseigenschaften etc. beizubringen.

### Produktinformationsdatei und Notifizierung kosmetischer Fertigprodukte

Jedes Unternehmen muss sicherstellen, dass sein kosmetisches Fertigprodukt für den Verbraucher sicher ist. Für jedes Produkt ist eine Produktinformationsdatei (PID) zu führen. Diese enthält umfassende Informationen zu allen Inhaltsstoffen und deren Änderungen, Meldungen, Berichte und Bewertungen unerwünschter Verbraucherereignisse sowie regelmäßig erstellte Produktsicherheitsberichte. Die PID kann Gegenstand von Audits durch die EU-Behörden sein. Der Verkauf eines Sonnenschutzmittels ist zulässig, nachdem das Unternehmen die EU-Behörde über das Meldeportal benachrichtigt hat [6]. Fertigerzeugnisse, die Nanomaterialien enthalten, müssen 6 Monate vor dem Inverkehrbringen gemeldet werden. Ein Katalog von Nanomaterialien, die in Kosmetika auf dem EU-Markt sind, kann eingesehen werden [3].

## Gefahr versus Risiko

Die unklare Wahrnehmung von Gefahren und Risiken hat sicherlich zur Verunsicherung und Angst vor Nanopartikeln in Sonnenschutzmitteln beigetragen – und das, obwohl Menschen in manchen Alltagssituationen sehr wohl zwischen Gefahr und Risiko unterscheiden können. Wenn man die Straße überquert, hat das Auto das Potenzial, Schäden zu verursachen. Daher stellt das Auto an sich eine Gefahr dar. Das Risiko ist die Wahrscheinlichkeit eines Schadens aufgrund der Exposition. Wenn man eine stark befahrene Autobahn oder einen wenig befahrenen Feldweg überquert, ist das Risiko eines Schadens hoch bzw. gering. Wenn man dieses Beispiel auf die Situation mit Nanopartikeln anwendet, ist die Situation ähnlich!

Risiko ist die Wahrscheinlichkeit eines Schadens aufgrund der Exposition

Aufgrund einiger einzigartiger physikochemischer Eigenschaften (Größe, Oberfläche, Reaktivität) haben Nanopartikel das Potenzial, Schäden zu verursachen, und stellen daher eine Gefahr dar. Wenn die Lunge Nanopartikeln ausgesetzt wird, ist das Risiko (Wahrscheinlichkeit) einer Schädigung hoch, während es bei einer Exposition der Haut sehr gering ist. Das Lungengewebe verfügt nur über begrenzte Mechanismen, um das Ein- und Durchdringen von Nanopartikeln zu verhindern. Im Gegensatz dazu ist die Haut mit ihrem Stratum corneum von Natur aus ideal geeignet, um eben genau dies zu verhindern. Die Abb. 2 und 3 zeigen deutlich, dass unlösliche Filter – aufgrund ihrer Größe – kaum ins Stratum corneum eindringen können.

Unlösliche UV-Filter können das Stratum corneum nicht durchdringen

Lösliche Filter hingegen sind in der Lage, das Stratum corneum in beträchtlichen Mengen zu überwinden, wie wiederholt nachgewiesen wurde [14, 15]. Daher ist das Risiko negativer Auswirkungen infolge der Absorption (systemische Bioverfügbarkeit) bei löslichen Filtern höher als bei unlöslichen Filtern.

## Science-Fiction-Geschichten, wissenschaftliche Veröffentlichungen und Medien

Wie der Zauberlehrling in der gleichnamigen Ballade von Johann Wolfgang von Goethe haben alte und neue Science-Fiction-Geschichten sowie die Medien ein Gespenst geschaffen, von dem wir uns kaum befreien können. Die Angst vor den kleinen Partikeln, die durch die Haut vom Blut aufgenommen und im ganzen Körper verteilt werden und Krankheiten verursachen, ist allgegenwärtig. Begriffe wie Nanobots oder „Nano, das neue Asbest“ haben sich zu eigenständigen Bildern entwickelt, die Ängste vermitteln. Auch Wissenschaftler verwenden das Vokabular von Science-Fiction-Geschichten wie die Metapher der „Nanocontainer“, die einen Wirkstoff (z. B. Zytostatikum) an bestimmte Zielorte (z. B. Tumor) im Körper transportieren und abgeben. Diesen Narrativen kann nur mit differenzierter Darstellung dokumentierter Faktenlagen in verständlicher Sprache unter Vermeidung von Metaphern begegnet werden.

## Quo vadis?

Es ist nachvollziehbar, dass der Begriff „Nano“ als eine Metapher für besonders klein wahrgenommen wird. Gleichzeitig muss man sich aber vor Augen führen, dass nanopartikuläre (unlösliche) UV-Filter größer sind als alle löslichen Filter. Die Darstellung der anatomischen Verhältnisse in der Hautbarriere und die Größenverhältnisse von nanopartikulären (unlöslichen) und löslichen Filtern sind ein klarer Hinweis, dass keine signifikante Absorption von nanopartikulären (unlöslichen) Filtern stattfinden kann. Daher sind nanopartikuläre (unlösliche) Filter eine Alternative zu löslichen Filtern.

In den letzten 30 Jahren haben 6 partikuläre Filter eine EU-Zulassungen bzw. eine Scientific Committee on Consumer Safety(SCCS)-Stellungnahme erhalten. Parallel dazu haben sich in Europa eine moderne Gesetzgebung und Zulassungspraxis für UV-Filter einschließlich deren Nanoformen entwickelt. Die Zulassungen derzeit verfügbarer nanopartikulärer Filter basieren auf strengen Gefährdungs- und Risikobewertungen. Alle nanopartikulären Filter haben aktualisierte und/oder neue Zulassungen bzw. wissenschaftliche Stellungnahmen.

Nanopartikuläre UV-Filter sind wesentlich größer als alle löslichen Filter

Fachkräfte im Gesundheitswesen und Wissenschaftler sollten die Öffentlichkeit auf deren Verständnisniveau informieren und v. a. vermeiden, Metaphern zu verwenden, die bekannte Ängste bedienen. Nicht zuletzt sollte auch die Presse korrekt informieren und jedwede Sensationalisierung vermeiden.

## Fazit für die Praxis

- Nanopartikuläre UV-Filter sind wesentlich größer als alle löslichen Filter. Eine perkutane Absorption solcher Filter ist nicht möglich. Produkte mit nanopartikulären Filtern sind für Kinder, schwangere Frauen, stillende Mütter und Außenarbeiter, die täglich über lange Zeiträume topischen Sonnenschutz anwenden, geeignet.
- Die Begrifflichkeit Gefahr, Risiko und Exposition wird oft nicht klar kommuniziert. Aufgrund einzigartiger physikochemischer Eigenschaften haben Nanopartikel das Potenzial, Schäden zu verursachen, und können daher eine Gefahr darstellen. Wenn die Lunge Nanopartikeln ausgesetzt wird, ist das Risiko (Wahrscheinlichkeit) einer Schädigung hoch, während es bei einer Exposition der Haut sehr gering ist. **Das Lungengewebe verfügt über begrenzte Mechanismen, um das Eindringen von Nanopartikeln zu verhindern. Im Gegensatz dazu ist die Haut mit ihrem Stratum corneum von Natur aus ideal geeignet, um eben gerade dies zu verhindern.**
- Fachkräfte im Gesundheitswesen und die Presse sollten die Öffentlichkeit auf deren Verständnisniveau informieren und vermeiden, Metaphern zu verwenden, die bekannte Ängste rund um „Nano“ bedienen.
