# Erratum zu: Nano ist groß! Fakten und Mythen über nanopartikuläre UV-Filter

**DOI:** 10.1007/s00105-022-04981-y

**Published:** 2022-03-11

**Authors:** Christian Surber, James Plautz, Uli Osterwalder

**Affiliations:** 1 Dermatologische Klinik, UniversitätsSpital Zürich, Gloriastr. 31, 8091 Zürich, Schweiz; 2 Dermatologische Klinik, Universitätsspital Basel, Petersgraben 4, 4031 Basel, Schweiz; 3 CHRYSALIS Services AG, Bäumleingasse 10, 4051 Basel, Schweiz; 4 Sun Protection Facilitator GmbH, Pfeffingerstr. 82, 4053 Basel, Schweiz

**Erratum zu:**

**Hautarzt 2022**

10.1007/s00105-022-04954-1

Im Originalbeitrag war Tab. 1 leider fehlerhaft:

Die Filterkonzentration von Tris-biphenyl Triazine (nano) (TBPT) mEk ist **10** **%**.

Die korrigierte Tabelle finden Sie im Folgenden.

**Table 1:** 

*Anorganische UV-Filter,* International Nomenclature of Cosmetic Ingredients (INCI)
– Titanium Dioxide (nano), maximale Einsatzkonzentration (mEk) 25 % [24]
– Zinc Oxide (nano) mEk 25 % [23]
*Organische UV-Filter,* INCI
– Methylene Bis-benzotriazolyl Tetramethylbutylphenole (nano) (MBBT) mEk 10 % [25]
– Tris-biphenyl Triazine (nano) (TBPT) mEk 10 % [22]
– Phenylene Bis-diphenyl Triazine (PBDT) mEk 5 % [19]
– Bis-(Diethylaminohydroxybenzoyl Benzoyl) Piperazine (nano) (HAA299) mEk 10 % [20]

Der Originalbeitrag wurde korrigiert.

